# Supplementary material for: Refinement of a meaning-centered counseling program for Chinese patients with advanced cancer: integrating cultural adaptation and implementation science approaches
Source: BMC Health Serv Res. 2025 Jan 15;25:85. doi: 10.1186/s12913-024-12124-3 (PMC11736934; doi:10.1186/s12913-024-12124-3)
Supplement: Supplementary file 1 — Supplementary Material 1. [file 12913_2024_12124_MOESM1_ESM.docx]

**Supplemental File 1.** Mental Health Professionals Survey and Semi-Structured Interview Guide – Refinement and Adaptation of Meaning-Centered Psychotherapy for Chinese Patients with Advanced Cancer.

**survey**

**Section I: DemographiC information & professional experience**

1. **gender**

( ) Male ( ) Female

1. **what is your race or ethnic origin?**

( ) Chinese

( ) White/Caucasian

( ) Black/African American

( ) Other Asian

*Please specify_______________*

( ) Some other race

*Please specify_______________*

1. **what is your country of birth?**

( ) United States

( ) China

( ) Taiwan

( ) Hong Kong

( ) Other, please specify: _____________________

1. **if not born in the united states, in what year did you move to the united states?**

Year: _________

( ) Don’t know

( ) N/A

( ) Refused

1. **how well do you speak english?**

( ) Very well

( ) Well

( ) Not well

( ) Not at all well

( ) Prefer not to answer

1. **how well do you speak mandarin chinese?**

( ) Very well

( ) Well

( ) Not well

( ) Not at all well

( ) Prefer not to answer

1. **how well do you read simplified chinese?**

( ) Very well

( ) Well

( ) Not well

( ) Not at all well

( ) Prefer not to answer

1. **what is your dominant language?**

( ) English

( ) Mandarin Chinese

( ) Other, please specify

( ) Prefer not to answer

1. **in general, what language(s) do you read and speak?**

( ) Chinese is better than English

( ) English is better than Chinese

( ) Both equally

( ) Prefer not to answer

1. **what educational degree do you hold?**

( ) Master degree in psychology

( ) Master degree in counseling

( ) Master degree in social work

( ) PhD in psychology

( ) PsyD in psychology

( ) PhD in counseling

( ) PhD in social work

( ) MD, Psychiatry

( ) MD, Other, please specify: _____________________

( ) Advanced nursing degree

( ) Graduate student in training

( ) Other, please specify: _____________________

( ) Prefer not to answer

1. **in what country did you receive your degree?** _____________________
2. **in what year did you receive your degree?** _____________________
3. **my primary job is:**

( ) Mental health professional

( ) Health professional

( ) Academic professor/educator

( ) Palliative care professional

( ) Researcher

( ) Other, please specify: _____________________

( ) Prefer not to answer

1. **in what year were you licensed?**

( ) Please specify year: _____________________

( ) N/A, not licensed

1. **do you provide counseling, psychotherapy, or mental health services in chinese?**

( ) Yes

( ) No

( ) Prefer not to answer

1. **do you provide counseling, psychotherapy, or mental health services to adults, children, or adolescents?**

(  ) Adults only

(  ) Children and adolescents only

(  ) Children, adolescents, and adults

(  ) Prefer not to answer

1. **how many years have you spent in clinical practice since you got your degree?**

(  ) Less than a year

(  ) 1-2 years

(  ) 3-5 years

(  ) 6-10 years

(  ) 11-15 years

(  ) 16-20 years

( ) More than 20 years

(  ) Prefer not to answer

1. **on average, how many hours do you spend providing counseling, psychotherapy, or mental health services?**

(  ) Less than 5 hours

(  ) 5-10 hours

(  ) 11-20 hours

(  ) 21-30 hours

(  ) 31-40 hours

(  ) More than 40 hours

(  ) Prefer not to answer

1. **on average, how many clients do you see per week?**

(  ) Less than 5 clients/patients

(  ) 5-10 clients/patients

(  ) 11-20 clients/patients

(  ) 21-30 clients/patients

(  ) 31-40 clients/patients

(  ) 41-50 clients/patients

(  ) More than 50 clients/patients

(  ) Prefer not to answer

1. **on average, how many cancer patients (or survivors) do you see per week?**

(  ) None

( ) Less than 5 clients/patients

(  ) 5-10 clients/patients

(  ) 11-20 clients/patients

(  ) 21-30 clients/patients

(  ) 31-40 clients/patients

(  ) More than 50 clients/patients

(  ) Prefer not to answer

1. **on average, how many *chinese* cancer patients (or survivors) do you see per week?**

(  ) None

( ) Less than 5 clients/patients

(  ) 5-10 clients/patients

(  ) 11-20 clients/patients

(  ) 21-30 clients/patients

(  ) 31-40 clients/patients

(  ) More than 40 clients/patients

(  ) Prefer not to answer

1. **where do you provide counseling, psychotherapy, or mental health services to chinese patients? (check all that apply)**

(  ) Private office

( ) Mental health clinic

(  ) Cancer clinic or hospital

(  ) Private organization

(  ) Academic institution

(  ) Other, please specify: ___________________

(  ) Prefer not to answer

1. **on average, how many advanced (stages iii or iv) cancer patients do you see per week?**

(  ) None

( ) Less than 5 clients/patients

(  ) 5-10 clients/patients

(  ) 11-20 clients/patients

(  ) 21-30 clients/patients

(  ) 31-40 clients/patients

(  ) More than 40 clients/patients

(  ) Prefer not to answer

1. **how comfortable are you working with chinese patients?**

(  ) Very uncomfortable

( ) Uncomfortable

(  ) Neutral

(  ) Comfortable

(  ) Very comfortable

( ) N/A; I have not worked with Chinese cancer patients

(  ) Prefer not to answer

1. **how comfortable are you working with chinese cancer patients?**

(  ) Very uncomfortable

( ) Uncomfortable

(  ) Neutral

(  ) Comfortable

(  ) Very comfortable

( ) N/A; I have not worked with Chinese cancer patients

(  ) Prefer not to answer

1. **how comfortable are you working with patients with advanced (stages iii or iv) cancer?**

(  ) Very uncomfortable

( ) Uncomfortable

(  ) Neutral

(  ) Comfortable

(  ) Very comfortable

( ) N/A; I have not worked with patients with advanced cancer

(  ) Prefer not to answer

1. **how many formal courses or seminars have you taken on cultural competency in the areas of psychotherapy, multicultural counseling, or any other related training?**

(  ) Very uncomfortable

( ) Uncomfortable

(  ) Neutral

(  ) Comfortable

(  ) Very comfortable

( ) N/A; I have not worked with patients with advanced cancer

(  ) Prefer not to answer

1. **how many formal health psychology, psychosomatic, or behavioral medicine courses did you take during your formal training program (md/ma/phd, etc.)?**

(  ) 0

( ) 1

(  ) 2

(  ) More than 2, please specify # ___

1. **how many formal cancer-related, psycho-oncology or supportive oncology courses, workshops, seminars, or conferences have you taken/attended?**

(  ) 0

( ) 1

(  ) 2

(  ) More than 2, please specify # ___

1. **have you ever attended a conference or meeting organized by these professional organizations? (check all that apply)**

(  ) American Psycho-Oncology Society

( ) Chinese Psychosocial Oncology Society

(  ) American Society of Clinical Oncology

(  ) Taiwanese Psycho-Oncology Society

(  ) Society of Behavioral Medicine

( ) Asian American Psychological Association

(  ) Prefer not to answer

**SECTION II: FEEDBACK ON MCP-CH SAMPLE SCRIPTS**

Instructions: Below are selected scripts from all six sessions of the Meaning Centered Program for Chinese Patients with Advanced Cancer (MCP-Ch). Please rate the scripts on 1) relevance to the target population (Chinese patients with advanced cancer); 2) writing style; 3) language appropriateness (appropriate for the target population, considering literacy/educational level); 4) culturally acceptable (cultural relevance and acceptability). The answers are 1) high, 2) moderate or suboptimal (needs modification) and 3) low (content or language is inappropriate, requires extensive modification).

| Section | **SESSION 1: CONCEPTS AND SOURCES OF MEANING** | |
| --- | --- | --- |
| 1 | **Intervention Introduction**  **Script Example**  **Therapist:** Welcome to your first session of the Meaning Centered Program. This program is based on the work of Viktor Frankl, the author of *Man’s Search for Meaning* and a number of other books on the subject of finding a sense of meaning in life. Don’t worry if you have not read this book yet. This program is tailored for Chinese cancer patients, so we will work together to explore meaning in life within the unique context of Chinese culture. We will meet weekly for a total of six sessions. In these sessions, we will discuss the concept of meaning and how people find a sense of meaning and purpose in their life, both in general and after the cancer diagnosis. Terms like meaning and purpose can be rather vague and can mean different things to different people, so we will include some exercises as well as a lot of discussion of the concepts that come up and how they relate to your own experiences and those of cancer patients. | 1. Do you think that the language used (i.e. “Meaning Centered Program” instead of “psychotherapy) is appropriate for the population, considering culture and literacy/education level?   ( ) Yes, highly appropriate  ( ) Moderately appropriate, needs minor modifications  ( ) Not appropriate, the language needs extensive modification   1. Do you think that the metaphors/stories used (if any) are appropriate for the population, considering culture and literacy/education level?   ( ) Yes, highly appropriate  ( ) Moderately appropriate, needs minor modifications  ( ) Not appropriate, the metaphors/stories need extensive modification  ( ) N/A, no metaphors or stories used in this section   1. Do you think the goals of this script are relevant to Chinese patients with advanced cancer?   ( ) Yes, high relevance  ( ) Moderate relevance, needs minor modifications  ( ) Low relevance, the goals are inappropriate and need extensive modification   1. Do you think the content is appropriate and culturally relevant for Chinese patients with advanced cancer?   ( ) Yes, highly appropriate  ( ) Moderately appropriate, needs minor modifications  ( ) Not appropriate, the content needs extensive modification   1. Do you think the concepts (i.e. “meaning”) are appropriate and culturally relevant for Chinese patients with advanced cancer?   ( ) Yes, highly appropriate  ( ) Moderately appropriate, needs minor modifications  ( ) Not appropriate, the concepts need extensive modification  If you chose moderate or not appropriate for any of the above questions, please elaborate below: |
| 2 | **Patient’s Cancer History**  **Script Example**  **Therapist:** Today we will start with introductions to each other and to the program in general, as well as a brief introduction to concepts and sources of meaning. In general, we will start each session with a check-in regarding how you are doing personally and medically and then briefly describe the topics that will be explored in the session. The rest of the session will focus on relevant exercises and open discussions related to topics focused on meaning. Sometimes, there will be homework or topics to think about in between sessions. One way to explore the idea of meaning is to read Frankl’s book, *Man’s Search for Meaning*. We have provided you with a copy that has been translated into Chinese. Feel free to read the book at a pace that is comfortable to you.  *The therapist should briefly introduce him/herself (e.g. name, credentials, staff position). Then encourage the patient to briefly introduce him/herself:*   - Full name - Place of birth - If you are married or have children - Your diagnosis - Date of your diagnosis   What has the doctor told you about your condition and treatment? Is your cancer curable or not?  [*If the patient seems hesitant to share their cancer story, the therapist may model for the patient by sharing an example:*  **Therapist:** It might not be easy for you to share your cancer story with me. I will share with you the cancer story of one of our study participants, who was diagnosed with Stage IV breast cancer. This might make it easier for you to tell me your story:  “I had a very full life … I was working 15-16 hours a day. I have two children. They’re adults and they’re 32 and soon to be 27. When I was diagnosed with metastatic breast cancer I immediately went to the computer to figure out what that was. Cause it didn’t make sense to me that you could be diagnosed with stage IV breast cancer and not have gone through stages I, II, and III. It just didn’t seem logical … I approached my illness as life changing … I [realized] my life can’t continue as it was, it just has to change. And where the focus was on work, I changed the focus to my family. My daughter was getting married that year. I asked everyone to pray for me that I would live long enough to see her married.” This patient discussed how having cancer made her change her life and priorities, to change her focus from work to family. What is your cancer story?] | 1. Do you think that the language used is appropriate for the population, considering culture and literacy/education level?   ( ) Yes, highly appropriate  ( ) Moderately appropriate, needs minor modifications  ( ) Not appropriate, the language needs extensive modification   1. Do you think that the metaphors/stories used (if any) are appropriate for the population, considering culture and literacy/education level?   ( ) Yes, highly appropriate  ( ) Moderately appropriate, needs minor modifications  ( ) Not appropriate, the metaphors/stories need extensive modification  ( ) N/A, no metaphors or stories used in this section   1. Do you think the goals of this script are relevant to Chinese patients with advanced cancer?   ( ) Yes, high relevance  ( ) Moderate relevance, needs minor modifications  ( ) Low relevance, the goals are inappropriate and need extensive modification   1. Do you think the content is appropriate and culturally relevant for Chinese patients with advanced cancer?   ( ) Yes, highly appropriate  ( ) Moderately appropriate, needs minor modifications  ( ) Not appropriate, the content needs extensive modification   1. Do you think the concepts (i.e. “sources of meaning”) are appropriate and culturally relevant for Chinese patients with advanced cancer?   ( ) Yes, highly appropriate  ( ) Moderately appropriate, needs minor modifications  ( ) Not appropriate, the concepts need extensive modification  If you chose moderate or not appropriate for any of the above questions, please elaborate below: |
| 3 | **Patient’s Definition of Meaning**  **Script Example**  **Therapist:**  Now let's talk about the definition of meaning. What does meaning mean to you?  [Wait for the patient’s definition]  [*If the patient is uncertain, the therapist may model for the patient by sharing an example:*  **Therapist:** There is no right or wrong answer to this question. The definition of meaning is greatly dependent on one’s personal experience. With patients’ consent, we conducted interviews on adapting this meaning-centered program for Chinese patients with advanced cancer. In one of our interviews, a cancer patient shared with us, “Before I got cancer, I felt that no matter if it’s at work or in my family, I did a good job. I was very independent. I could rely on myself. I had financial independence. I felt I was living a meaningful life. But, once I got sick, especially after the doctor told me that I had only a few months to live, the meaning of my life changed. Now that I don’t know how much longer I can live, I don’t care about these things as much as I did before. I thought, as long as I live for one more day, I should make everyone around me happy. That’s what is meaningful to me now.” So, it sounds like this patient changed his definition of meaning after having cancer. What are your thoughts on this? How would you define meaning?]  Very well, for you, meaning means [repeat patient’s definition].  However, the definition for us and how we will be using it for this therapy is, first:  **Having the idea that one's life has meaning means knowing that one is fulfilling a unique purpose in life and that life is a gift.**   1. A life that comes with the responsibility of living our potential to the fullest 2. By doing so, we are able to achieve a sense of peace, tranquility and even transcendence through the connection with something greater than oneself.   And second: **Meaning refers to the moments that make life worth living, when one feels alive or needed. It can also include things from the past that, when you look back, you still think are very important.**  Could you tell me, using your own words, what you understood by meaning?  [If the patient understood] Very good  [If the patient did not understand] It seems to me that what you understood was [Repeat what the patient understood]. Again, what we refer to by meaning is [Repeat the definition until you confirm that the patient has understood]  **Sheet #1**  **Definition of Meaning**   1. **Having the idea that one's life has meaning means knowing that one is fulfilling a unique purpose in life and that life is a gift.** 2. A life that comes with the responsibility of living our potential to the fullest. 3. By doing so we are able to achieve a sense of peace, tranquility and even transcendence, through the connection with something greater than oneself. 4. **Meaning refers to the moments that make life worth living, when one feels alive or needed. It can also include things from the past that when you look back you still think are very important.** | 1. Do you think that the language used is appropriate for the population, considering culture and literacy/education level?   ( ) Yes, highly appropriate  ( ) Moderately appropriate, needs minor modifications  ( ) Not appropriate, the language needs extensive modification   1. Do you think that the metaphors/stories used (if any) are appropriate for the population, considering culture and literacy/education level?   ( ) Yes, highly appropriate  ( ) Moderately appropriate, needs minor modifications  ( ) Not appropriate, the metaphors/stories need extensive modification  ( ) N/A, no metaphors or stories used in this section   1. Do you think the goals of this script are relevant to Chinese patients with advanced cancer?   ( ) Yes, high relevance  ( ) Moderate relevance, needs minor modifications  ( ) Low relevance, the goals are inappropriate and need extensive modification   1. Do you think the content is appropriate and culturally relevant for Chinese patients with advanced cancer?   ( ) Yes, highly appropriate  ( ) Moderately appropriate, needs minor modifications  ( ) Not appropriate, the content needs extensive modification   1. Do you think the concepts (i.e. “a unique purpose in life,” “transcendence through connection with something greater than oneself”) are appropriate and culturally relevant for Chinese patients with advanced cancer?   ( ) Yes, highly appropriate  ( ) Moderately appropriate, needs minor modifications  ( ) Not appropriate, the concepts need extensive modification  If you chose moderate or not appropriate for any of the above questions, please elaborate below: |
| 4 | **Overview of Session Topics**  **Sheet #2**  **Structured Weekly Topics for the Meaning Centered Program**  **Section #1**: Concepts and Sources of Meaning  **Section #2**: Cancer and Meaning  **Section #3**: Meaning from Your Personal Story – Past, Present, and Future  **Section #4**: Meaning Shaped by Your Attitude  **Section #5**: Meaning through Creativity and Experience  **Section #6**: Transition: Reflections and Hopes for the Future  **Script Example**  **Therapist:** Session 1 will focus on the "Concepts and Sources of Meaning", both in general and in terms of how meaning can relate specifically to cancer. Sources of meaning refer to where our meaning comes from, to what is significant in our lives. Session 2 will focus on "Cancer and Meaning," and emphasize how you may see yourself differently before and after your cancer diagnosis. Session 3 will explore your personal story in the past, present, and future. We will explore how the things you were taught, your upbringing, family, children, friends, relationships, job, and traditions and values make you who you are today. Session 4 will address how your attitude can shape your story. Do you still find meaning in your daily life even though you know there are limits? What gets in the way of finding meaning? How can we address these difficulties? Session 5 will explore meaning of life through creativity and in-the-moment experience. How can we create meaning in the things we spend time doing, such as work, being a parent or caregiver, or participating in one’s community? How do we create meaning through experiencing love, beauty, and even humor? This could include spending time with loved ones, observing nature when taking a walk, or listening to music. The final session will give us a chance to reflect upon your experiences in this meaning-centered program, as well as to explore your hopes for the future. | 1. Do you think that the language used is appropriate for the population, considering culture and literacy/education level?   ( ) Yes, highly appropriate  ( ) Moderately appropriate, needs minor modifications  ( ) Not appropriate, the language needs extensive modification   1. Do you think that the metaphors/stories used (if any) are appropriate for the population, considering culture and literacy/education level?   ( ) Yes, highly appropriate  ( ) Moderately appropriate, needs minor modifications  ( ) Not appropriate, the metaphors/stories need extensive modification  ( ) N/A, no metaphors or stories used in this section   1. Do you think the goals of this script are relevant to Chinese patients with advanced cancer?   ( ) Yes, high relevance  ( ) Moderate relevance, needs minor modifications  ( ) Low relevance, the goals are inappropriate and need extensive modification   1. Do you think the content is appropriate and culturally relevant for Chinese patients with advanced cancer?   ( ) Yes, highly appropriate  ( ) Moderately appropriate, needs minor modifications  ( ) Not appropriate, the content needs extensive modification   1. Do you think the concepts (i.e. meaning through attitude, creativity, and experience) are appropriate and culturally relevant for Chinese patients with advanced cancer?   ( ) Yes, highly appropriate  ( ) Moderately appropriate, needs minor modifications  ( ) Not appropriate, the concepts need extensive modification  If you chose moderate or not appropriate for any of the above questions, please elaborate below: |
| 5 | **Introduction to Victor Frankl’s Meaning Centered Work:**  **Sheet #3**  ***“He who has a why to live for can bear with almost any how”***  **Script Example**  **Therapist:** A central theme of Frankl’s work is that the desire to find meaning in our existence is a primary motivating force in all human beings. He believed that life has meaning and never ceases to have meaning up until the last moment, though what’s meaningful may sometimes change over time as people’s circumstances change. While there are many aspects of suffering that we have no control over, Frankl suggests that we still have some freedom to consider and choose our attitude toward that suffering, even in as unlikely a place as a concentration camp, which he experienced firsthand for a number of years. | 1. Do you think that the language used is appropriate for the population, considering culture and literacy/education level?   ( ) Yes, highly appropriate  ( ) Moderately appropriate, needs minor modifications  ( ) Not appropriate, the language needs extensive modification   1. Do you think that the metaphors/stories used (if any) are appropriate for the population, considering culture and literacy/education level?   ( ) Yes, highly appropriate  ( ) Moderately appropriate, needs minor modifications  ( ) Not appropriate, the metaphors/stories need extensive modification  ( ) N/A, no metaphors or stories used in this section   1. Do you think the goals of this script are relevant to Chinese patients with advanced cancer?   ( ) Yes, high relevance  ( ) Moderate relevance, needs minor modifications  ( ) Low relevance, the goals are inappropriate and need extensive modification   1. Do you think the content is appropriate and culturally relevant for Chinese patients with advanced cancer?   ( ) Yes, highly appropriate  ( ) Moderately appropriate, needs minor modifications  ( ) Not appropriate, the content needs extensive modification   1. Do you think the concepts (i.e. “freedom to choose our attitude,” suffering as meaningful) are appropriate and culturally relevant for Chinese patients with advanced cancer?   ( ) Yes, highly appropriate  ( ) Moderately appropriate, needs minor modifications  ( ) Not appropriate, the concepts need extensive modification  If you chose moderate or not appropriate for any of the above questions, please elaborate below: |
| 6 | **Sources (Origins) of Meaning: What Do We Mean By “Meaning”?**  **Sheet #4**  **Meaning from Your Personal Story**   - Reviewing your past, present, and future - E.g. family history and upbringing, what you were taught; past and ongoing accomplishments (work, children, relationships with family, friends); your traditions and values; what you hope to leave behind, pass on   **Meaning Shaped by Your Attitude**   - How your attitude can shape your story: Do you still find meaning in your life even though you know there are limits? What gets in the way of finding meaning? How are you dealing with it? - E.g. overcoming physical pain/grief/loss with a positive attitude, enabling you to still find meaning in daily life   **Meaning through Creativity**   - How can we create meaning in the things we spend time doing, such as work, being a parent, or community activities? - E.g. finding meaning in volunteer work or helping other cancer patients   **Meaning through Experience**   - Creating meaning through experiencing love and beauty (or even humor) - E.g. spending time with loved ones, observing nature when taking a walk, or listening to music   **Script Example**  **Therapist:** The phrase “sources of meaning” refers to the origins of meaning or to what is meaningful in our lives. Meaning can come from one’s personal story, personal attitude, creativity and daily life experiences (Refer to Sheet #4 above).  **Meaning from Your Personal Story**. Meaning is given through our story and history. For example, what are your traditions and values? How does your family history shape what is important to you? What lessons do you hope to leave behind and pass on to friends, family, or future generations?  **Meaning Shaped by Your Attitude**: Finding meaning through attitude refers to the attitude one assumes when faced with life’s limitations. For example, personal hardship, physical pain, or emotional suffering. We can choose our attitude toward suffering or limitations. Sometimes a tragedy can turn into a personal triumph.  **Meaning through Creativity**: Creativity includes our roles and responsibilities, such as work, being a parent, or community activities. How do we find meaning in the things we spend time doing?  **Meaning through Experience**: Meaning through life experiences includes connecting to life through relationships (loved ones), through beauty in nature or art, or even through humor. | 1. Do you think that the language used is appropriate for the population, considering culture and literacy/education level?   ( ) Yes, highly appropriate  ( ) Moderately appropriate, needs minor modifications  ( ) Not appropriate, the language needs extensive modification   1. Do you think that the metaphors/stories used (if any) are appropriate for the population, considering culture and literacy/education level?   ( ) Yes, highly appropriate  ( ) Moderately appropriate, needs minor modifications  ( ) Not appropriate, the metaphors/stories need extensive modification  ( ) N/A, no metaphors or stories used in this section   1. Do you think the goals of this script are relevant to Chinese patients with advanced cancer?   ( ) Yes, high relevance  ( ) Moderate relevance, needs minor modifications  ( ) Low relevance, the goals are inappropriate and need extensive modification   1. Do you think the content is appropriate and culturally relevant for Chinese patients with advanced cancer?   ( ) Yes, highly appropriate  ( ) Moderately appropriate, needs minor modifications  ( ) Not appropriate, the content needs extensive modification   1. Do you think the concepts (i.e. “meaning from your personal story,” “meaning shaped by your attitude,” “meaning through creativity,” and “meaning through experience”) are appropriate and culturally relevant for Chinese patients with advanced cancer?   ( ) Yes, highly appropriate  ( ) Moderately appropriate, needs minor modifications  ( ) Not appropriate, the concepts need extensive modification  If you chose moderate or not appropriate for any of the above questions, please elaborate below: |
| 7 | **Experiential Exercise**  **Sheet #5:**  **Session #1 Experiential Exercise - Sources of Meaning**  Now that we have talked about meaning and the sources of meaning, I ask you:  *List one or two experiences or moments in your life that you have felt have been especially meaningful for you (regardless of whether it sounds deep or trivial). For example, something that helps you cope with a difficult day; or a moment when you feel more alive. And say something about it.*  **Script Example**  [*If the patient is hesitant, the therapist may model for the patient by sharing an example:*  **Therapist:** You may be processing your memories. I will give you an example from one of our previous patients, who shared with us, “I couldn’t have children of my own due to my liver cancer. My niece, who’s 7, means the world to me. One of the most meaningful moments was being able to swim with her, and be her playmate, and have her run into my arms and hug me. I love to feel her energy and her love and her enthusiasm and her positivity. I was enveloped in it. We also had so much pleasure drinking in nature. Nature is so spiritual and so meaningful for me. So being outdoors, and connecting with my niece, and with nature, it’s just been such a gift … And then, because I had a pretty good attitude when I went back [to the doctor’s office for follow-up visits] after my liver transplant, I began to talk to patients who have liver cancer like me. Thanks to our shared experience of having undergone a serious operation, there were certain things I was able to say to them about the bumpy road and about the healing process. I am not a nurse or a doctor, but now, when I go to see my doctor, I usually try to take an afternoon and see patients who are having problems pre- or post- transplant. Talking with and helping people make me feel good, and these have also become meaningful moments for me.”  This is an example shared by one of our previous patients. She later summarized that recalling these meaningful moments allowed her to acknowledge three sources of meaning: meaning through experience, such as playing with her niece over the summer and getting pleasure in nature, being outdoors, and connecting with her niece; meaning shaped by attitude; in that her positive attitude despite her difficult circumstances and focus on helping others is what enabled her to volunteer and share her experience with other cancer patients; and meaning through creativity, where she found meaning through the volunteer work she was doing visiting and helping other cancer patients like herself. Can you share with me one or two of your most meaningful moments?] | 1. Do you think that the language used is appropriate for the population, considering culture and literacy/education level?   ( ) Yes, highly appropriate  ( ) Moderately appropriate, needs minor modifications  ( ) Not appropriate, the language needs extensive modification   1. Do you think that the metaphors/stories used (if any) are appropriate for the population, considering culture and literacy/education level?   ( ) Yes, highly appropriate  ( ) Moderately appropriate, needs minor modifications  ( ) Not appropriate, the metaphors/stories need extensive modification  ( ) N/A, no metaphors or stories used in this section   1. Do you think the goals of this script are relevant to Chinese patients with advanced cancer?   ( ) Yes, high relevance  ( ) Moderate relevance, needs minor modifications  ( ) Low relevance, the goals are inappropriate and need extensive modification   1. Do you think the content is appropriate and culturally relevant for Chinese patients with advanced cancer?   ( ) Yes, highly appropriate  ( ) Moderately appropriate, needs minor modifications  ( ) Not appropriate, the content needs extensive modification   1. Do you think the concepts (i.e. “meaningful moments in life”) are appropriate and culturally relevant for Chinese patients with advanced cancer?   ( ) Yes, highly appropriate  ( ) Moderately appropriate, needs minor modifications  ( ) Not appropriate, the concepts need extensive modification  If you chose moderate or not appropriate for any of the above questions, please elaborate below: |

| Section | **SESSION 2: CANCER AND MEANING** | |
| --- | --- | --- |
| 8 | **Introduction to Session #2: Cancer and Meaning**  **Script Example**  **Therapist:** Suffering occurs any time we, as humans, confront a limitation. Cancer presents many such limitations. Suffering can include physical pain, emotional pain, and spiritual pain. In times of stress, people may feel that they lose meaning, value or purpose in their life. As Frankl has shown through his experiences in the concentration camp, we have the ability to find meaning in suffering and in life itself. We can make use of the sources of meaning (refer to "Sources of Meaning" - Sheet # 4). By choosing our attitude towards suffering, we can learn to find, maintain, or even improve, meaning and purpose in our life even as we face limitations, including a cancer experience. | 1. Do you think that the language used is appropriate for the population, considering culture and literacy/education level?   ( ) Yes, highly appropriate  ( ) Moderately appropriate, needs minor modifications  ( ) Not appropriate, the language needs extensive modification   1. Do you think that the metaphors/stories used (if any) are appropriate for the population, considering culture and literacy/education level?   ( ) Yes, highly appropriate  ( ) Moderately appropriate, needs minor modifications  ( ) Not appropriate, the metaphors/stories need extensive modification  ( ) N/A, no metaphors or stories used in this section   1. Do you think the goals of this script are relevant to Chinese patients with advanced cancer?   ( ) Yes, high relevance  ( ) Moderate relevance, needs minor modifications  ( ) Low relevance, the goals are inappropriate and need extensive modification   1. Do you think the content is appropriate and culturally relevant for Chinese patients with advanced cancer?   ( ) Yes, highly appropriate  ( ) Moderately appropriate, needs minor modifications  ( ) Not appropriate, the content needs extensive modification   1. Do you think the concepts (i.e. “finding meaning in suffering”) are appropriate and culturally relevant for Chinese patients with advanced cancer?   ( ) Yes, highly appropriate  ( ) Moderately appropriate, needs minor modifications  ( ) Not appropriate, the concepts need extensive modification  If you chose moderate or not appropriate for any of the above questions, please elaborate below: |
| 9 | **Experiential Exercise: Identity and Cancer**  **Script Example**  **Therapist:** In the following exercise we will talk about your identity and how cancer has had an impact on your identity.  First, what does identity mean to you?  [Wait for the patient’s definition]  Very well, for you, identity means [repeat the patient’s definition]. However, for us it means: **One’s identity is made up of roles, relationships, traditions, beliefs, and values. For example, parent or caregiver, your religious or spiritual beliefs. These are the sources of meaning in one's life. To reflect on the sources of meaning in each person's life, it is important to start with your own understanding of who you are.**  Could you tell me, using your own words, what you understood by identity?  [If the patient understood] Very good  [Or if the patient did not understand] It seems to me that what you understood was [repeat what the patient understood]. Again, what we refer to as identity is [repeat the definition until you are certain that the patient has understood]  Now that we talked briefly about what we mean by identity, we are going to proceed with the exercise.  **Sheet #6**  **Session #2 Experiential Exercise – Identity and Cancer**  ***Identity before Cancer***  1. Answer this question from your perspective before your cancer diagnosis.  Write down four answers to the question:  "Who am I as a person?"  These can be positive or negative characteristics, including:  • Role in your family, job or community  • Personality characteristics,  • Body image,  • Beliefs,  • Things that you do,  • People you know, etc.  For example: I am someone who ... I am ...  ***Identity after Cancer***  2. Do you think that cancer has influenced your answers?  • Yes: How has it influenced the things that are most meaningful to you?  • No: Why do you think cancer has not had an impact?  *[If the patient is hesitant, the therapist may model for the patient by sharing an example:*  **Therapist:** There are many ways to answer this question. I will share with you a response from one of our study participants, who was diagnosed with metastatic stage IV breast cancer. This might make it easier for you to share. People describe their identities in all sorts of ways. “[Before cancer], I had a very long work schedule that pulled me away from the family … I used to joke at work a lot that on my death bed am I really going to think this was important? … When I heard the [cancer] diagnosis … I thought to myself, now that I’m really thinking about my deathbed, ‘How important is my work?’ … I have to put my family first … So it was for me, a refocusing of my life. My life became my family.” So, it sounds like this patient’s identity was centered around her family, and she made some changes to her life after her diagnosis. What changed for her? Can you describe how your identity has remained the same or changed after cancer?] | 1. Do you think that the language used is appropriate for the population, considering culture and literacy/education level?   ( ) Yes, highly appropriate  ( ) Moderately appropriate, needs minor modifications  ( ) Not appropriate, the language needs extensive modification   1. Do you think that the metaphors/stories used (if any) are appropriate for the population, considering culture and literacy/education level?   ( ) Yes, highly appropriate  ( ) Moderately appropriate, needs minor modifications  ( ) Not appropriate, the metaphors/stories need extensive modification  ( ) N/A, no metaphors or stories used in this section   1. Do you think the goals of this script are relevant to Chinese patients with advanced cancer?   ( ) Yes, high relevance  ( ) Moderate relevance, needs minor modifications  ( ) Low relevance, the goals are inappropriate and need extensive modification   1. Do you think the content is appropriate and culturally relevant for Chinese patients with advanced cancer?   ( ) Yes, highly appropriate  ( ) Moderately appropriate, needs minor modifications  ( ) Not appropriate, the content needs extensive modification   1. Do you think the concepts (i.e. “cancer’s impact on identity”) are appropriate and culturally relevant for Chinese patients with advanced cancer?   ( ) Yes, highly appropriate  ( ) Moderately appropriate, needs minor modifications  ( ) Not appropriate, the concepts need extensive modification  If you chose moderate or not appropriate for any of the above questions, please elaborate below: |
| 10 | **Preview Session 3 and Invite Family Member to Join**  **Script Example**  **Therapist:** The next session will be on meaning from your personal story. We will explore how your traditions, family history, and values are sources of meaning in your life. We often find that people like to bring in a family member or loved one to this session. Would you like to do so? Why or why not?  [If patient would like to bring in a family member, caregiver, or loved one to the session, discuss logistics, including whether they will come for the full session or part of the session (patients may prefer to bring in a family member in the second half, when “Life as a Living Legacy that Will Be Passed On” is discussed), and what the patient would like to accomplish or convey to their loved one in the session.] | 1. Do you think that the language used is appropriate for the population, considering culture and literacy/education level?   ( ) Yes, highly appropriate  ( ) Moderately appropriate, needs minor modifications  ( ) Not appropriate, the language needs extensive modification   1. Do you think that the metaphors/stories used (if any) are appropriate for the population, considering culture and literacy/education level?   ( ) Yes, highly appropriate  ( ) Moderately appropriate, needs minor modifications  ( ) Not appropriate, the metaphors/stories need extensive modification  ( ) N/A, no metaphors or stories used in this section   1. Do you think the goals of this script are relevant to Chinese patients with advanced cancer?   ( ) Yes, high relevance  ( ) Moderate relevance, needs minor modifications  ( ) Low relevance, the goals are inappropriate and need extensive modification   1. Do you think the content is appropriate and culturally relevant for Chinese patients with advanced cancer?   ( ) Yes, highly appropriate  ( ) Moderately appropriate, needs minor modifications  ( ) Not appropriate, the content needs extensive modification   1. Do you think the concepts (i.e. “meaning from your personal story”) are appropriate and culturally relevant for Chinese patients with advanced cancer?   ( ) Yes, highly appropriate  ( ) Moderately appropriate, needs minor modifications  ( ) Not appropriate, the concepts need extensive modification  If you chose moderate or not appropriate for any of the above questions, please elaborate below: |

| Section | **SESSION 3: MEANING FROM YOUR PERSONAL STORY – PAST, PRESENT, AND FUTURE** | |
| --- | --- | --- |
| 11 | **Introduction to Session #3: Meaning from Your Personal Story – Past, Present, and Future**  **Script Example**  **Therapist:** Next, we are going to talk about your personal story and how cancer has impacted it.  But first, what does personal story mean to you?  [Wait for the patient’s definition]  Very well, for you, personal story means (repeat the patient’s definition). However, for us it means: **Every person has a past, a present and a future. This influences who we are. We receive a legacy from the past, a legacy that we experience and a legacy that we will pass on.**  Could you tell me in your own words what you understood by personal story?  [If the patient understood] Very good  [Or if the patient did not understand] It seems to me that what you understood was [repeat what the patient understood]. Again, what we refer to by personal story is [repeat the definition until you confirm that the patient has understood]  We mentioned the word legacy. Could you tell me what legacy means to you?  [Wait for the patient’s definition]  Very well. For you, legacy means [repeat the patient’s definition]. However, for us **legacy refers to 1) the inheritance of material and non-material things that we receive. Legacy could be biological/genetic, family, cultural heritage, family values, traditions, etc., 2) at the same time, we build a legacy through our lives, and 3) we will pass on a legacy, in the future, to our loved ones and to those who knew us.**  Could you tell me in your own words what you understood by legacy?  [If the patient understood] Very good  [Or if the patient did not understand] It seems to me that what you understood was [repeat what the patient understood]. Again, what we mean by legacy is [repeat the definition until you confirm that the patient has understood] | 1. Do you think that the language used is appropriate for the population, considering culture and literacy/education level?   ( ) Yes, highly appropriate  ( ) Moderately appropriate, needs minor modifications  ( ) Not appropriate, the language needs extensive modification   1. Do you think that the metaphors/stories used (if any) are appropriate for the population, considering culture and literacy/education level?   ( ) Yes, highly appropriate  ( ) Moderately appropriate, needs minor modifications  ( ) Not appropriate, the metaphors/stories need extensive modification  ( ) N/A, no metaphors or stories used in this section   1. Do you think the goals of this script are relevant to Chinese patients with advanced cancer?   ( ) Yes, high relevance  ( ) Moderate relevance, needs minor modifications  ( ) Low relevance, the goals are inappropriate and need extensive modification   1. Do you think the content is appropriate and culturally relevant for Chinese patients with advanced cancer?   ( ) Yes, highly appropriate  ( ) Moderately appropriate, needs minor modifications  ( ) Not appropriate, the content needs extensive modification   1. Do you think the concepts (i.e. “legacy”) are appropriate and culturally relevant for Chinese patients with advanced cancer?   ( ) Yes, highly appropriate  ( ) Moderately appropriate, needs minor modifications  ( ) Not appropriate, the concepts need extensive modification  If you chose moderate or not appropriate for any of the above questions, please elaborate below: |
| 12 | **Meaning in Our Past**  **Script Example**  **Therapist:** One of the characteristics that differentiate humans from animals is the fact that we have a sense of history, some knowledge of what came before us. This means that our lives have a history that includes values and traditions from our past. Our story is shaped by our history—our family history, immigration history, cultural history, etc. Part of your personal story is the legacy that your family and your past have given you, and part of your personal story is what you create. The story one creates, or the life one lives, may involve overcoming the past, or connecting to and building from it. Our personal story—what is sweet, sour bitter, and spicy in life? 酸甜苦辣 (*suan tian ku la*) Telling your story connects you to the people around you and keeps you connected to them, whether or not they are physically present. Thinking about the story of your life helps you reflect again on what you have found most meaningful, what tasks you have undertaken and what tasks are pending. These tasks can be in any area: Personal, social, family, work, etc. They can be personal stories to write, children to take care of, lessons to learn or teach, relationships to attend to, work, art, or volunteer projects, etc. One can even find meaning in the very act of telling the events of our lives. What is important about these activities is determining whether and how they are meaningful to you. I would like you to start telling your story in this session. How do you fit in with the story of your family, your traditions and values, your immigration history, your friendships, your community, etc.? So let's start exploring together.  **Sheet #7**  **Session #3 Experiential Exercise 1 – Life as a Given Legacy**  When you look back and think about your life, what are:  1) The most meaningful memories,  2) Relationships,  3) Traditions, etc., that have had the greatest impact on who you are today?  For example: Identify specific memories of how you grew up that have made a lasting impression on your life (for example, your relationships with grandparents, parents, children, siblings, friends, teachers, etc.)  Sometimes the origin of your name is a meaningful key to your family's legacy. What is the origin of your name? Does your last name link you to any meaningful historical figures or ancestors? What about your first name? | 1. Do you think that the language used is appropriate for the population, considering culture and literacy/education level?   ( ) Yes, highly appropriate  ( ) Moderately appropriate, needs minor modifications  ( ) Not appropriate, the language needs extensive modification   1. Do you think that the metaphors/stories used (if any) are appropriate for the population, considering culture and literacy/education level?   ( ) Yes, highly appropriate  ( ) Moderately appropriate, needs minor modifications  ( ) Not appropriate, the metaphors/stories need extensive modification  ( ) N/A, no metaphors or stories used in this section   1. Do you think the goals of this script are relevant to Chinese patients with advanced cancer?   ( ) Yes, high relevance  ( ) Moderate relevance, needs minor modifications  ( ) Low relevance, the goals are inappropriate and need extensive modification   1. Do you think the content is appropriate and culturally relevant for Chinese patients with advanced cancer?   ( ) Yes, highly appropriate  ( ) Moderately appropriate, needs minor modifications  ( ) Not appropriate, the content needs extensive modification   1. Do you think the concepts (i.e. “life as a given legacy”) are appropriate and culturally relevant for Chinese patients with advanced cancer?   ( ) Yes, highly appropriate  ( ) Moderately appropriate, needs minor modifications  ( ) Not appropriate, the concepts need extensive modification  If you chose moderate or not appropriate for any of the above questions, please elaborate below: |
| 13 | **Meaning in our Present and Future**  **Script Example**  **Therapist:** The "legacy we live" in the present is always changing. It includes meaningful roles, activities and achievements that have made life worth living. Examples include: the legacy of being an immigrant to offer a better life for your children, the legacy of being a father or grandfather, the legacy that one lives through work, church (*if the patient practices a religion*), or in the community (*re: volunteering or activities/special causes*). What is important to remember is that the life we live now creates the memories for the legacy we will pass on. So we must begin to evaluate how the "legacy we live" in the present could affect "the legacy we are going to pass on" in the future.  Meaningful questions arise: What are the life lessons we hope to transmit? How are we going to contribute to a greater whole? How will we be remembered? What will last beyond me?  **Sheet #8**  **Session #3 Experiential Exercise 2 – Life as a Living Legacy that Will Be Passed On**  By reflecting on who you are today, what are:  1) The activities,  2) roles,  3) or achievements (things you have done) that you feel most proud of?  When looking to the future, what are some of the life lessons that you would like to pass on to others?  What is the legacy that you would like to live and pass on?  *For patients who bring a loved one or family member to session, this exercise may be a meaningful one to undergo in the presence of a loved one. If appropriate, the therapist may direct the patient to speak to their loved one directly about “the legacy that you would like to live and pass on.”* | 1. Do you think that the language used is appropriate for the population, considering culture and literacy/education level?   ( ) Yes, highly appropriate  ( ) Moderately appropriate, needs minor modifications  ( ) Not appropriate, the language needs extensive modification   1. Do you think that the metaphors/stories used (if any) are appropriate for the population, considering culture and literacy/education level?   ( ) Yes, highly appropriate  ( ) Moderately appropriate, needs minor modifications  ( ) Not appropriate, the metaphors/stories need extensive modification  ( ) N/A, no metaphors or stories used in this section   1. Do you think the goals of this script are relevant to Chinese patients with advanced cancer?   ( ) Yes, high relevance  ( ) Moderate relevance, needs minor modifications  ( ) Low relevance, the goals are inappropriate and need extensive modification   1. Do you think the content is appropriate and culturally relevant for Chinese patients with advanced cancer?   ( ) Yes, highly appropriate  ( ) Moderately appropriate, needs minor modifications  ( ) Not appropriate, the content needs extensive modification   1. Do you think the concepts (i.e. “life as a living legacy that will be passed on”) are appropriate and culturally relevant for Chinese patients with advanced cancer?   ( ) Yes, highly appropriate  ( ) Moderately appropriate, needs minor modifications  ( ) Not appropriate, the concepts need extensive modification  If you chose moderate or not appropriate for any of the above questions, please elaborate below: |

| Section | **SESSION 4: MEANING SHAPED BY YOUR ATTITUDE** | |
| --- | --- | --- |
| 14 | **Introduction to Session #4: Meaning Shaped by Your Attitude**  **Script Example**  **Therapist:** During session 4 we will be focusing on the idea of choosing your own attitude.  But first, what does attitude mean to you?  [Wait for the patient’s definition]  Very well, for you, attitude means [repeat the patient’s definition]. However, for us it means: **Attitude is the ability (or freedom) to choose how we react to life situations.**  Could you tell me, using your own words, what you understood by attitude?  [If the patient understood] Very good  [Or if the patient did not understand] It seems to me that what you understood was (repeat what the patient understood). Again what we refer to by attitude is [repeat the definition until you confirm that the patient has understood] | 1. Do you think that the language used is appropriate for the population, considering culture and literacy/education level?   ( ) Yes, highly appropriate  ( ) Moderately appropriate, needs minor modifications  ( ) Not appropriate, the language needs extensive modification   1. Do you think that the metaphors/stories used (if any) are appropriate for the population, considering culture and literacy/education level?   ( ) Yes, highly appropriate  ( ) Moderately appropriate, needs minor modifications  ( ) Not appropriate, the metaphors/stories need extensive modification  ( ) N/A, no metaphors or stories used in this section   1. Do you think the goals of this script are relevant to Chinese patients with advanced cancer?   ( ) Yes, high relevance  ( ) Moderate relevance, needs minor modifications  ( ) Low relevance, the goals are inappropriate and need extensive modification   1. Do you think the content is appropriate and culturally relevant for Chinese patients with advanced cancer?   ( ) Yes, highly appropriate  ( ) Moderately appropriate, needs minor modifications  ( ) Not appropriate, the content needs extensive modification   1. Do you think the concepts (i.e. “meaning shaped by your attitude”) are appropriate and culturally relevant for Chinese patients with advanced cancer?   ( ) Yes, highly appropriate  ( ) Moderately appropriate, needs minor modifications  ( ) Not appropriate, the concepts need extensive modification  If you chose moderate or not appropriate for any of the above questions, please elaborate below: |
| 15 | **Confronting Life’s Limitations**  **Script Example**  **Therapist:** Let’s continue. Knowing that life is not forever (生老病死, *sheng lao bing si*, “you’re born, you get old, you get sick, and you die”) is largely the reason why we feel that our lives must have a meaning or purpose. Learning to handle limitations is what allows us to appreciate what we have. As Frankl says in *Man’s Search for Meaning*, "... often an exceptionally difficult external situation is what gives man the opportunity to grow spiritually beyond himself." Frankl considered his own experiences as proof of his inner strength. Facing such difficult situations brought him a feeling of pride and achievement.  The fact that we only have a limited time also challenges us to make the most of the time we have. For example, think about the materials that are required to build a house: Wood, nails, metals, beams, windows, doors. Imagine someone building their house. This person has a limited amount of time to finish their work. Not knowing when the deadline will be forces them to use time as best they can and to make the most of every moment. The wood we hammer is our life and the nails our values, memories, beliefs, things that we consider meaningful, and our attitude. Therefore, just as we cannot judge a meal or a dinner for the amount of food (but rather by how the food was prepared and by its taste), we cannot judge our lives simply by their length but by how we have lived it. This includes how we see life and death, how we face the limits and the losses, and by the meaning we find.  **Sheet #9**  **Session #4 Experiential Exercise 1 – Confronting Life’s Limitations**   1. What are some of the limitations, losses or obstacles that you have faced in the past, and how did you face them at that time? 2. Since your diagnosis, what are the limitations or the losses that you have faced, and how are you facing or dealing with these limitations or losses? Do you find meaning in your life despite knowing the limitations that life has and that life ends? [If yes, describe briefly.] 3. How do you imagine being remembered by your loved ones? For example, what are some of   a) your personal characteristics,  b) shared memories or  c) significant events in your life?  4) (OPTIONAL) What would you consider a peaceful death (安息的走, *an xin de zou,* “to die peacefully with no regret”) or meaningful death? What would you consider a “good” death (*bai xi shi*, “white happy event”)? | 1. Do you think that the language used is appropriate for the population, considering culture and literacy/education level?   ( ) Yes, highly appropriate  ( ) Moderately appropriate, needs minor modifications  ( ) Not appropriate, the language needs extensive modification   1. Do you think that the metaphors/stories used (if any) are appropriate for the population, considering culture and literacy/education level?   ( ) Yes, highly appropriate  ( ) Moderately appropriate, needs minor modifications  ( ) Not appropriate, the metaphors/stories need extensive modification  ( ) N/A, no metaphors or stories used in this section   1. Do you think the goals of this script are relevant to Chinese patients with advanced cancer?   ( ) Yes, high relevance  ( ) Moderate relevance, needs minor modifications  ( ) Low relevance, the goals are inappropriate and need extensive modification   1. Do you think the content is appropriate and culturally relevant for Chinese patients with advanced cancer?   ( ) Yes, highly appropriate  ( ) Moderately appropriate, needs minor modifications  ( ) Not appropriate, the content needs extensive modification   1. Do you think the concepts (i.e. “confronting life’s limitations”) are appropriate and culturally relevant for Chinese patients with advanced cancer?   ( ) Yes, highly appropriate  ( ) Moderately appropriate, needs minor modifications  ( ) Not appropriate, the concepts need extensive modification  If you chose moderate or not appropriate for any of the above questions, please elaborate below: |
| 16 | **“Legacy Project”: Assignment for Session Five**  **Script Example**  **Therapist:** We want to remind you of the theme, 'Life as a Legacy,' by creating your own “Legacy Project.” This is a project that you can undertake. It integrates some of the ideas that we have already discussed (meaning, traditions, values, responsibilities to family and loved ones) to generate a sense of meaning in light of your life and illness. Some examples include:  1) Keeping a diary or writing about your personal story  2) Creating a photo album  3) Making a video  4) Developing a music collection of meaningful songs  5) Creating a work of art (a painting, a sculpture, something in wood or clay)  6) Knitting a piece of clothing or blanket  7) Writing a recipe book  8) Creating and/or organizing an activity (family, fundraising for a cause, etc.)  9) Repairing a broken relationship; forgiving or asking for forgiveness  10) Undertaking something that you have always wanted to do and have not done yet... the legacy depends on you! | 1. Do you think that the language used is appropriate for the population, considering culture and literacy/education level?   ( ) Yes, highly appropriate  ( ) Moderately appropriate, needs minor modifications  ( ) Not appropriate, the language needs extensive modification   1. Do you think that the metaphors/stories used (if any) are appropriate for the population, considering culture and literacy/education level?   ( ) Yes, highly appropriate  ( ) Moderately appropriate, needs minor modifications  ( ) Not appropriate, the metaphors/stories need extensive modification  ( ) N/A, no metaphors or stories used in this section   1. Do you think the goals of this script are relevant to Chinese patients with advanced cancer?   ( ) Yes, high relevance  ( ) Moderate relevance, needs minor modifications  ( ) Low relevance, the goals are inappropriate and need extensive modification   1. Do you think the content is appropriate and culturally relevant for Chinese patients with advanced cancer?   ( ) Yes, highly appropriate  ( ) Moderately appropriate, needs minor modifications  ( ) Not appropriate, the content needs extensive modification   1. Do you think the concepts (i.e. “life as a legacy”, “legacy project”) are appropriate and culturally relevant for Chinese patients with advanced cancer?   ( ) Yes, highly appropriate  ( ) Moderately appropriate, needs minor modifications  ( ) Not appropriate, the concepts need extensive modification  If you chose moderate or not appropriate for any of the above questions, please elaborate below: |

| Section | **SESSION 5: MEANING THROUGH CREATIVITY AND EXPERIENCE** | |
| --- | --- | --- |
| 17 | **Introduction to Session #5: Meaning Through Creativity and Experience**  **Script Example**  **Therapist:** In this session we will focus on the idea of ​​finding meaning through creativity and experience.  But first, what does creativity mean to you?  [Wait for the patient’s definition]  Very well, for you, creativity means [repeat the patient’s definition]. However, for us creativity **refers to the act of creating in our lives. We shape our destiny through what we create. We can create material things, the meals we cook for our families, crafts, work products, etc. We can also create nonmaterial things. We create our family and our community. Living life and making decisions requires creativity.**  Could you tell me in your own words what you understood by creativity?  [If the patient understood] Very good  [Or if the patient did not understand] It seems to me that what you understood was [repeat what the patient understood]. Again, what we refer to by creativity is [repeat the definition until you confirm that the patient understood)]  Now, what does “being present” mean to you?  [Wait for the patient’s definition]  Very well, to you, “experience” means [repeat the patient’s definition]. However, to us **“experience” comes from experiencing life mainly through our senses and feelings. Experiencing life in the present through imagery, sound, taste, smell and touch, and emotion (happiness, amazement, laughter). For example, feelings you have in the moment while spending time with a loved one, or taking a walk in nature.**  Could you tell me in your own words what you understood by “experience”?  [If the patient understood] Very good  [Or if the patient did not understand] It seems to me that what you understood was [repeat what the patient understood]. Again what we mean by “experience” is [repeat the definition until you confirm that the patient has understood] | 1. Do you think that the language used is appropriate for the population, considering culture and literacy/education level?   ( ) Yes, highly appropriate  ( ) Moderately appropriate, needs minor modifications  ( ) Not appropriate, the language needs extensive modification   1. Do you think that the metaphors/stories used (if any) are appropriate for the population, considering culture and literacy/education level?   ( ) Yes, highly appropriate  ( ) Moderately appropriate, needs minor modifications  ( ) Not appropriate, the metaphors/stories need extensive modification  ( ) N/A, no metaphors or stories used in this section   1. Do you think the goals of this script are relevant to Chinese patients with advanced cancer?   ( ) Yes, high relevance  ( ) Moderate relevance, needs minor modifications  ( ) Low relevance, the goals are inappropriate and need extensive modification   1. Do you think the content is appropriate and culturally relevant for Chinese patients with advanced cancer?   ( ) Yes, highly appropriate  ( ) Moderately appropriate, needs minor modifications  ( ) Not appropriate, the content needs extensive modification   1. Do you think the concepts (i.e. “meaning through creativity,” “meaning through experience”) are appropriate and culturally relevant for Chinese patients with advanced cancer?   ( ) Yes, highly appropriate  ( ) Moderately appropriate, needs minor modifications  ( ) Not appropriate, the concepts need extensive modification  If you chose moderate or not appropriate for any of the above questions, please elaborate below: |
| 18 | **Meaning Through Creativity**  **Script Example**  **Therapist:** Dr. Frankl (1955) suggested that **"we should not ask ourselves what to expect from life, but understand that life expects something from us.”** Life lays out its problems, and it is up to us to respond to these questions in a meaningful way.  The task of creating a meaningful life is incredibly challenging. As human beings we are imperfect and many times we do not live to our full unique potential. We experience this as imperfection, vulnerability, and shortcomings. We may sometimes feel we have not done “enough.”  The beauty of creativity is that it continually gives us a second chance to start over, amend, forge new roads, traverse unexplored territories and transcend our limits. The challenge of creativity is that it takes a lot of courage, tenacity and inner strength to continually risk facing uncertainty and doubt. **"The value is not the absence of fear, it is, rather, the ability to move forward despite it"** (May, 1975, p.12). It takes a great deal of courage to face an advanced diagnosis of cancer and find the energy and inner determination to move forward despite an uncertain future.  **Sheet #10**  **Session #5 Experiential Exercise 1 - Actively Engaging in Life**  **Creativity, Courage and Responsibility**   1. Living life requires courage and responsibility.    1. Could you give me an example in your life in which you were brave?    2. Could you give me an example in your life in which you assumed a significant responsibility?    3. Could you give me an example in your life in which you made a significant or valuable commitment? 2. Do you feel that you have expressed what is most meaningful to you through your creative activities? How? (for example, through work, parenting or caregiving, hobbies, causes) 3. What are your responsibilities? To whom or to what are you responsible? 4. Do you have things or wishes that you have not been able to achieve yet? For example, a goal, a project, unresolved conflicts, etc. If so, what is stopping you?? | 1. Do you think that the language used is appropriate for the population, considering culture and literacy/education level?   ( ) Yes, highly appropriate  ( ) Moderately appropriate, needs minor modifications  ( ) Not appropriate, the language needs extensive modification   1. Do you think that the metaphors/stories used (if any) are appropriate for the population, considering culture and literacy/education level?   ( ) Yes, highly appropriate  ( ) Moderately appropriate, needs minor modifications  ( ) Not appropriate, the metaphors/stories need extensive modification  ( ) N/A, no metaphors or stories used in this section   1. Do you think the goals of this script are relevant to Chinese patients with advanced cancer?   ( ) Yes, high relevance  ( ) Moderate relevance, needs minor modifications  ( ) Low relevance, the goals are inappropriate and need extensive modification   1. Do you think the content is appropriate and culturally relevant for Chinese patients with advanced cancer?   ( ) Yes, highly appropriate  ( ) Moderately appropriate, needs minor modifications  ( ) Not appropriate, the content needs extensive modification   1. Do you think the concepts (i.e. “creativity, courage, and responsibility”) are appropriate and culturally relevant for Chinese patients with advanced cancer?   ( ) Yes, highly appropriate  ( ) Moderately appropriate, needs minor modifications  ( ) Not appropriate, the concepts need extensive modification  If you chose moderate or not appropriate for any of the above questions, please elaborate below: |
| 19 | **Meaning Through Experience**  **Script Example**  **Therapist:** Meaning through experience is often experienced in a more passive way in contrast to the other more active sources of meaning, such as creativity and attitude. You can think of this as “being” vs. “doing.” When one surrenders to the feeling of love, being in nature, the taste of a delicious meal, or experiencing art or beauty, one can feel more present in life itself. The sources of meaning derived from experience - love, nature, beauty, connecting with God (*for religious patients*), listening to music, or even humor - allow us to feel a part of something bigger than ourselves, like the waves of water that come together to form the ocean. We can find nature, the beauty of the mountains, the ocean or a sunset particularly more intensely during adverse circumstances. For example, imagine Zhang Wei, an immigrant who has traveled far from his country and has not been able to return for many years despite wanting to for a long time. The beauty of his native country, his town or his city, would be all the more meaningful if this person could go back. We can also feel transported by love, even when the loved one is not physically present.  **Sheet #11**  **Session #5- Experiential Exercise 2 – Connecting to Life**  List three ways in which you feel more alive and connected to life through the experience of:  Love:   1. __________________ 2. __________________ 3. __________________   Nature:   1. __________________ 2. __________________ 3. __________________   Other (Religion, Beauty, Art, Music, Humor, etc.):   1. __________________ 2. __________________ 3. __________________ | 1. Do you think that the language used is appropriate for the population, considering culture and literacy/education level?   ( ) Yes, highly appropriate  ( ) Moderately appropriate, needs minor modifications  ( ) Not appropriate, the language needs extensive modification   1. Do you think that the metaphors/stories used (if any) are appropriate for the population, considering culture and literacy/education level?   ( ) Yes, highly appropriate  ( ) Moderately appropriate, needs minor modifications  ( ) Not appropriate, the metaphors/stories need extensive modification  ( ) N/A, no metaphors or stories used in this section   1. Do you think the goals of this script are relevant to Chinese patients with advanced cancer?   ( ) Yes, high relevance  ( ) Moderate relevance, needs minor modifications  ( ) Low relevance, the goals are inappropriate and need extensive modification   1. Do you think the content is appropriate and culturally relevant for Chinese patients with advanced cancer?   ( ) Yes, highly appropriate  ( ) Moderately appropriate, needs minor modifications  ( ) Not appropriate, the content needs extensive modification   1. Do you think the concepts (i.e. “connecting to life through love, nature, religion, beauty, art, music, and humor”) are appropriate and culturally relevant for Chinese patients with advanced cancer?   ( ) Yes, highly appropriate  ( ) Moderately appropriate, needs minor modifications  ( ) Not appropriate, the concepts need extensive modification  If you chose moderate or not appropriate for any of the above questions, please elaborate below: |

| Section | **SESSION 6: TRANSITIONS** | |
| --- | --- | --- |
| 20 | **Process Transitions**  **Script Example**  **Therapist:** Welcome to our final session of the Meaning Centered Program. Let’s talk about the work we have done over the last five sessions. [*Refer to* **Sheet #2 Structured Weekly Topics for the Meaning Centered Program** *and draw attention to the progression of the session’s topics to the final session today, identifying and emphasizing the weekly themes in the process.*] Have you reflected on what the last session might be like? Do you have any thoughts or feelings about ending our sessions and completing this program? [The discussion on 'transitions' should last about 30 minutes, depending on the patient's interest] | 1. Do you think that the language used is appropriate for the population, considering culture and literacy/education level?   ( ) Yes, highly appropriate  ( ) Moderately appropriate, needs minor modifications  ( ) Not appropriate, the language needs extensive modification   1. Do you think that the metaphors/stories used (if any) are appropriate for the population, considering culture and literacy/education level?   ( ) Yes, highly appropriate  ( ) Moderately appropriate, needs minor modifications  ( ) Not appropriate, the metaphors/stories need extensive modification  ( ) N/A, no metaphors or stories used in this section   1. Do you think the goals of this script are relevant to Chinese patients with advanced cancer?   ( ) Yes, high relevance  ( ) Moderate relevance, needs minor modifications  ( ) Low relevance, the goals are inappropriate and need extensive modification   1. Do you think the content is appropriate and culturally relevant for Chinese patients with advanced cancer?   ( ) Yes, highly appropriate  ( ) Moderately appropriate, needs minor modifications  ( ) Not appropriate, the content needs extensive modification   1. Do you think the concepts (i.e. “transitions”) are appropriate and culturally relevant for Chinese patients with advanced cancer?   ( ) Yes, highly appropriate  ( ) Moderately appropriate, needs minor modifications  ( ) Not appropriate, the concepts need extensive modification  If you chose moderate or not appropriate for any of the above questions, please elaborate below: |
| 21 | **Legacy Project Review**  [The therapist should smoothly transition from discussing endings to exploring new beginnings through the patient’s Legacy Project. Some patients may have chosen not to participate in this effort (due to lack of energy, interest, time, illness, etc.), so leave 15-20 minutes to explore the project (if it has been completed).] | 1. Do you think that the language used is appropriate for the population, considering culture and literacy/education level?   ( ) Yes, highly appropriate  ( ) Moderately appropriate, needs minor modifications  ( ) Not appropriate, the language needs extensive modification   1. Do you think that the metaphors/stories used (if any) are appropriate for the population, considering culture and literacy/education level?   ( ) Yes, highly appropriate  ( ) Moderately appropriate, needs minor modifications  ( ) Not appropriate, the metaphors/stories need extensive modification  ( ) N/A, no metaphors or stories used in this section   1. Do you think the goals of this script are relevant to Chinese patients with advanced cancer?   ( ) Yes, high relevance  ( ) Moderate relevance, needs minor modifications  ( ) Low relevance, the goals are inappropriate and need extensive modification   1. Do you think the content is appropriate and culturally relevant for Chinese patients with advanced cancer?   ( ) Yes, highly appropriate  ( ) Moderately appropriate, needs minor modifications  ( ) Not appropriate, the content needs extensive modification   1. Do you think the concepts (i.e. legacy project as “a new beginning”) are appropriate and culturally relevant for Chinese patients with advanced cancer?   ( ) Yes, highly appropriate  ( ) Moderately appropriate, needs minor modifications  ( ) Not appropriate, the concepts need extensive modification  If you chose moderate or not appropriate for any of the above questions, please elaborate below: |
| 22 | **Feedback about the Meaning Centered Program**  **Sheet #12**  **Session #6 Experiential Exercise - Experience: Reflection and Conclusion**   1. How has it been for you to go through this learning experience during these six sessions? Has there been any change in the way you see life and the cancer experience? 2. Do you feel that you have a better understanding about the sources of meaning and that you can use them in your daily life? If so, how? 3. What are your hopes for the future? | 1. Do you think that the language used is appropriate for the population, considering culture and literacy/education level?   ( ) Yes, highly appropriate  ( ) Moderately appropriate, needs minor modifications  ( ) Not appropriate, the language needs extensive modification   1. Do you think that the metaphors/stories used (if any) are appropriate for the population, considering culture and literacy/education level?   ( ) Yes, highly appropriate  ( ) Moderately appropriate, needs minor modifications  ( ) Not appropriate, the metaphors/stories need extensive modification  ( ) N/A, no metaphors or stories used in this section   1. Do you think the goals of this script are relevant to Chinese patients with advanced cancer?   ( ) Yes, high relevance  ( ) Moderate relevance, needs minor modifications  ( ) Low relevance, the goals are inappropriate and need extensive modification   1. Do you think the content is appropriate and culturally relevant for Chinese patients with advanced cancer?   ( ) Yes, highly appropriate  ( ) Moderately appropriate, needs minor modifications  ( ) Not appropriate, the content needs extensive modification   1. Do you think the concepts (i.e. “giving feedback”) are appropriate and culturally relevant for Chinese patients with advanced cancer?   ( ) Yes, highly appropriate  ( ) Moderately appropriate, needs minor modifications  ( ) Not appropriate, the concepts need extensive modification  If you chose moderate or not appropriate for any of the above questions, please elaborate below: |

**SECTION III: FEEDBACK ON PRISM PRE-IMPLEMENTATION FACTORS**

Instructions: The following questions concern factors that may affect the implementation of Meaning Centered Program for Chinese Immigrants with Advanced Cancer (MCP-Ch) in usual care settings. MCP-Ch is adapted from Meaning Centered Psychotherapy, a manualized intervention designed to meet the psychosocial needs of patients with advanced cancer. It is drawn from Victor Frankl’s logotherapy, which posits that individuals’ sense of meaning and purpose in life can be enhanced by helping them to connect to sources of meaning in their lives such as valued relationships, experiences, work, and activities. MCP is aimed at enhancing meaning in life, which is thought to drive improvements in spiritual well-being, quality of life, depression, anxiety, and hopelessness. In our study, MCP-Ch will be delivered remotely via telehealth (either by videoconference or by telephone, depending on patient preference) by a bilingual (English and Mandarin-speaking) therapist.

|  | **Question / PRISM Domains** | **Strongly Agree** | **Agree** | **Neutral** | **Disagree** | **Strongly Disagree** |
| --- | --- | --- | --- | --- | --- | --- |
| **Intervention Characteristics – Organizational Perspective** | | | | | | |
| 1 | I would use MCP-Ch in my work with Chinese patients with advanced cancer |  |  |  |  |  |
| 2 | I believe there is good evidence to support a meaning-centered approach to meet the psychosocial needs of Chinese patients with advanced cancer |  |  |  |  |  |
| 3 | My theoretical background and training lends itself well to using MCP-Ch with patients |  |  |  |  |  |
| 4 | I believe telehealth-delivered mental health interventions are safe and effective |  |  |  |  |  |
| 5 | I believe mental health interventions such as MCP-Ch can be delivered remotely to Chinese-speaking patients by monolingual English-speaking therapists via interpreters |  |  |  |  |  |
| 6 | Based upon my experience with the patient population, I believe Chinese patients with advanced cancer would find MCP-Ch helpful and acceptable |  |  |  |  |  |
| 7 | Based upon my experience with the patient population, I believe Chinese patients with advanced cancer would find telehealth delivery helpful and acceptable |  |  |  |  |  |
| 8 | Based upon my experience with the patient population, I believe Chinese patients with advanced cancer would find an interpreter-assisted, remotely delivered counseling program acceptable |  |  |  |  |  |
| **Recipients** | | | | | | |
| **Organizational Characteristics** | | | | | | |
| 9 | MCP-Ch could be feasibly utilized in my practice setting |  |  |  |  |  |
| 10 | My supervisor/administration would support my use of MCP-Ch |  |  |  |  |  |
| **Patient Characteristics** | | | | | | |
| 11 | My patients’ psychosocial needs (accounting for patient characteristics such as income, education level, acculturation level, cancer type, and current medical and/or mental health treatment) would be well met by MCP-Ch |  |  |  |  |  |
| **Implementation and Sustainability Infrastructure** | | | | | | |
| 10 | Implementing MCP-Ch in my current practice setting is feasible |  |  |  |  |  |
| 11 | I have the resources and training I need to use MCP-Ch in my current practice setting |  |  |  |  |  |
| **External Environment** | | | | | | |
| 12 | The current state and federal regulatory environment is conducive to delivering MCP-Ch via telehealth |  |  |  |  |  |
| 13 | The policies of my practice setting do not conflict with my use of MCP-Ch in clinical practice |  |  |  |  |  |

**survey**

**SECTION I: INTRODUCTORY COMMENTS**

The purpose of this interview is to collect information about the adaptations needed for the Meaning Centered Program for Chinese Patients with Advanced Cancer Manual. You already completed a structured assessment of the key sections. For the purposes of this interview, we will review the responses for which your rating was of low or moderate appropriateness or relevance. Our interpreter and trained linguist, Yunshan Niu, will provide interpretation remotely as needed.

Before we begin, I would like to remind you of your rights as a participant.

- Your answers will be kept anonymous and confidential – your identity will not be linked to any information about you and it will be available only to the researchers working on this study. Everyone’s answers will be combined together.
- There are no right or wrong answers, so you should respond by giving the answer that best describes your own experience. You can refuse or skip any questions, but your answers to other questions are very important to us.
- This interview will be video and audio recorded. Only the members of the research team will have access to the recordings or transcriptions. The information obtained through the interviews will be used for research purposes only.
- You can decide to stop at any time. Let us know if you are thinking about stopping or decide to stop. There are no consequences for stopping.
- There are no costs for taking part in this study. After the interview, you will receive $100 for your time.
- You also have the choice to not take part in this research study. We will notify you in the future about new information or changes in the study that may affect your willingness to continue in the study.

**Do you have any questions about this study and our discussion so far?**

You have received an explanation about the purpose of this study and you have consented. Please remember to avoid mentioning your name or the name of others since this conversation is going to be recorded.

**SECTION II: PROBING LOW/MODERATE RATINGS OF MCP-CH SAMPLE SCRIPTS**

I have your survey responses here. I would like to go over any responses indicating moderate or low appropriateness or relevance so that I can get your expert opinion on how to modify the language, metaphors, goals, content, and/or concepts to ensure the intervention is linguistically and culturally appropriate for the target population.

| **Sections** | **Rating (moderate or low)** | **If moderate or low, please elaborate** |
| --- | --- | --- |
| Section 1 | Language:  Metaphors/stories:  Goals:  Content:  Concepts: |  |
| Section 2 | Language:  Metaphors/stories:  Goals:  Content:  Concepts: |  |
| Section 3 | Language:  Metaphors/stories:  Goals:  Content:  Concepts: |  |
| Section 4 | Language:  Metaphors/stories:  Goals:  Content:  Concepts: |  |
| Section 5 | Language:  Metaphors/stories:  Goals:  Content:  Concepts: |  |
| Section 6 | Language:  Metaphors/stories:  Goals:  Content:  Concepts: |  |
| Section 7 | Language:  Metaphors/stories:  Goals:  Content:  Concepts: |  |
| Section 8 | Language:  Metaphors/stories:  Goals:  Content:  Concepts: |  |
| Section 9 | Language:  Metaphors/stories:  Goals:  Content:  Concepts: |  |
| Section 10 | Language:  Metaphors/stories:  Goals:  Content:  Concepts: |  |
| Section 11 | Language:  Metaphors/stories:  Goals:  Content:  Concepts: |  |
| Section 12 | Language:  Metaphors/stories:  Goals:  Content:  Concepts: |  |
| Section 13 | Language:  Metaphors/stories:  Goals:  Content:  Concepts: |  |
| Section 14 | Language:  Metaphors/stories:  Goals:  Content:  Concepts: |  |
| Section 15 | Language:  Metaphors/stories:  Goals:  Content:  Concepts: |  |
| Section 16 | Language:  Metaphors/stories:  Goals:  Content:  Concepts: |  |
| Section 17 | Language:  Metaphors/stories:  Goals:  Content:  Concepts: |  |
| Section 18 | Language:  Metaphors/stories:  Goals:  Content:  Concepts: |  |
| Section 19 | Language:  Metaphors/stories:  Goals:  Content:  Concepts: |  |
| Section 20 | Language:  Metaphors/stories:  Goals:  Content:  Concepts: |  |
| Section 21 | Language:  Metaphors/stories:  Goals:  Content:  Concepts: |  |
| Section 22 | Language:  Metaphors/stories:  Goals:  Content:  Concepts: |  |

**SECTION III: PROBING RESPONSES TO PRISM PRE-IMPLEMENTATION FACTORS**

I have your survey responses here. I would like to go over any responses in which you indicated “neutral,” “disagree,” or “strongly disagree” so that I can get more information on facilitators of and barriers to implementing MCP-Ch in real-world settings such as your clinical practice.

| **Question** | **Rating** | **Sample Probes** |
| --- | --- | --- |
| 1 | Neutral  Disagree  Strongly Disagree | Why not? What would keep you from using MCP-Ch? |
| 2 | Neutral  Disagree  Strongly Disagree | Why not? What kind of evidence would you like to see before using a meaning-centered approach with this population? |
| 3 | Neutral  Disagree  Strongly Disagree | Why not? What theoretical background or training is needed to support the use of MCP-Ch? |
| 4 | Neutral  Disagree  Strongly Disagree | Why not? What evidence or changes would you need to see to consider telehealth-delivered mental health interventions safe and effective? |
| 5 | Neutral  Disagree  Strongly Disagree | Why not? What changes to MCP-Ch would make it more helpful and acceptable to Chinese patients with advanced cancer? |
| 6 | Neutral  Disagree  Strongly Disagree | Why not? What adjustments could we make (i.e. providing mobile devices with data packages or providing technical support) to make telehealth delivery acceptable to Chinese patients with advanced cancer? |
| 7 | Neutral  Disagree  Strongly Disagree | Why not? What are your organization’s barriers to using MCP-Ch? |
| 8 | Neutral  Disagree  Strongly Disagree | Why not? What would need to change for leadership/administration to support the use of MCP-Ch? |
| 9 | Neutral  Disagree  Strongly Disagree | Why not? Which patient characteristics/demographics present barriers to using MCP-Ch? What modifications could we make to the intervention to better address their needs? |
| 10 | Neutral  Disagree  Strongly Disagree | Why not? What adaptations would make it feasible to use MCP-Ch in your practice setting? |
| 11 | Neutral  Disagree  Strongly Disagree | Why not? What resources (i.e., trained medical interpreters, telehealth resources) or training experiences do you need to use MCP-Ch in your practice setting? |
| 12 | Neutral  Disagree  Strongly Disagree | Why not? What are the regulatory barriers to delivering MCP-Ch via telehealth? How might they be addressed? |
| 13 | Neutral  Disagree  Strongly Disagree | Why not? What organizational policies present barriers to using MCP-Ch in clinical practice? How might they be addressed? |

Thank you for your participation!
